# Hemoglobin drop following postpartum hemorrhage

**DOI:** 10.1038/s41598-020-77799-0

**Published:** 2020-12-09

**Authors:** Enav Yefet, Avishag Yossef, Abeer Suleiman, Aliza Hatokay, Zohar Nachum

**Affiliations:** 1 Department of Obstetrics and Gynecology, Emek Medical Center, Yitzhak Rabin Boulevard 21, Afula, 1834111 Israel; 2 Department of Obstetrics and Gynecology, Baruch Padeh Medical Center Poriya, Tiberias, Israel; 3 Azrieli Faculty of Medicine, Bar-Ilan University, Safed, Israel; 4 Rappaport Faculty of Medicine, Technion, Haifa, Israel; 5 Department of Obstetrics and Gynecology, Holy Family Hospital, Nazareth, Israel

**Keywords:** Diseases, Health care, Medical research, Signs and symptoms

## Abstract

Postpartum hemorrhage (PPH) is defined as blood loss of ≥ 500–1000 ml within 24 h after delivery. Yet, assessment of blood loss is imprecise. The present study aimed to profile the hemoglobin (Hb) drop after vaginal delivery with versus without PPH. This was a secondary analysis of a prospective cohort study of women who delivered vaginally. Women were included if complete blood counts (CBC) before and after delivery were taken until stabilization (N = 419). Women were categorized into the PPH group and controls, for whom post-delivery CBCs were performed due to indications unrelated to bleeding. The PPH patients were then classified as either overt or occult PPH (symptoms related to hypovolemia without overt bleeding) subgroups. The primary endpoint was mean Hb drop after delivery. One hundred and ten (26%) and 158 (38%) women presented with overt PPH or occult PPH, respectively; 151 (36%) women were included in the control group. Mean Hb decrease from baseline was 3.0 ± 1.6, 2.0 ± 1.4 and 0.9 ± 1.0 g/dl, respectively (p < 0.0001). In all groups, maximal rate of Hb decline was in the first 6–12 h postpartum and plateaued after 24–48 h. At 48 h post-delivery, 95% and 86% of women who had dropped to Hb ≤ 9.5 and < 7 g/dl, respectively, reached those thresholds. Taken together, an Hb decrease ≥ 2 g/dl was consistent with PPH diagnosis and should be followed for at least 48 h after delivery.

## Introduction

Postpartum hemorrhage (PPH) is defined as a loss of ≥ 500–1000 ml blood from the genital tract, accompanied by signs or symptoms of hypovolemia within 24 h after the birth process^1,2^. It is the most common form of major obstetric hemorrhage and is associated with substantial mortality and morbidity rates. History of PPH is a risk factor for PPH in subsequent pregnancies and preventive measures are recommended^1^. Thus, PPH diagnosis should be as accurate as possible. Yet, PPH definition and follow-up is based on subjective gross estimations of blood loss, which are often imprecise and tend to underestimate blood loss, consequentially delaying diagnosis^3–6^. In one study, almost 20% of women with severe PPH, defined by fulfillment of at least one of the following criteria: transfusion of red blood cell concentrates, conservative surgery for PPH, arterial embolization, hysterectomy, transfer to intensive care unit due to PPH, death attributed to PPH, or hemoglobin (Hb) decrease by > 4 g/dl, were diagnosed solely on the basis of laboratory tests^7^.

Hypothetically, a drop in Hb may provide an objective measure for PPH diagnosis. Indeed, it is customary to perform a complete blood count (CBC) during PPH events for baseline measures. However, guidelines regarding normal and abnormal ranges and durations of Hb drop after delivery remain elusive. Studies reporting on these parameters examined therapies or other markers for overt PPH, and excluded women without PPH or with symptoms related to hypovolemia (occult PPH) without overt bleeding.

The present study aimed to elucidate Hb and hematocrit (HCT) patterns in women following vaginal delivery without PPH, with occult PPH (defined as symptoms related to anemia/hypovolemia without a report on overt hemorrhage) or with overt PPH. The study also aimed to assess the duration of follow-up required to identify and treat PPH and postpartum anemia.

## Methods

### Design

This was a secondary analysis of data collected in a prospective cohort trial which assessed the efficacy of a routine screening protocol for postpartum anemia diagnosis and treatment in the maternity ward of women who delivered vaginally. The study evaluated two protocols for postpartum anemia detection^8^.

The study was conducted between June 29, 2015 and January 27, 2016, at Emek Medical Center, a university-affiliated hospital in Israel (ClinicalTrials.gov identifier: NCT02434653, date of registration: 28/04/2015). The study was approved by the local review board of the Emek Medical Center (EMC 112-14) and was performed in accordance with relevant guidelines and regulations of the institutional review board. Participants provided signed informed consent.

Women who intended to or eventually delivered vaginally (spontaneous or by vacuum extraction) were assessed for eligibility at the labor and delivery, maternal fetal medicine, or maternity wards. Inclusion criteria were women above 18 years of age who delivered vaginally and who had undergone CBC tests postpartum. Women who had known allergy to iron sucrose were not eligible to participate in the study. In addition, women with pre-eclampsia with severe features were excluded from the original study, which was designed to evaluate a screening protocol for postpartum anemia and, according to the departmental protocol, women with pre-eclampsia with severe features are routinely screened by taking a Hb test every 8 h.

In this study we divided women meeting the eligibility criteria with PPH into one of two groups:

1. Overt PPH—Women with estimated blood loss of ≥ 500 ml or hemorrhage accompanied by symptoms of hypovolemia.

2. Occult PPH—Women with symptomatic anemia without the presence of hemorrhage at the time of delivery.

CBCs were performed prior to or immediately after delivery. After delivery, additional postpartum CBC tests were performed in cases consistent with either overt PPH (according to estimated blood loss), on presentation of signs and symptoms of anemia/hypovolemia (occult PPH) or in cases of severe anemia before delivery (Hb < 8 g/dl). In women recruited after October 11, 2015, additional CBC tests were also performed in cases of peri-delivery anemia (i.e., Hb < 10.5 g/dl), regardless of symptoms. Additional CBC tests were performed at the physician’s discretion (e.g., maternal fever and assessment of pre-eclampsia).

CBC tests were performed until Hb stabilized, defined as < 1 g/dl decrease between two tests at a minimal interval of 8 h. Tests were performed earlier, according to the physician’s discretion, in cases of substantial or active bleeding, severe symptoms consistent with hemorrhage or anemia, or hemodynamic instability.

Women reporting on anemia-related symptoms or overt bleeding underwent a thorough physical examination, including position and tone of the uterus, determined by abdominal examination, and vaginal examination for the presence of blood clots, tears and hematomas. Pelvic ultrasound was also performed to assess the uterine cavity as well as pelvic and abdominal fluid, according to physical findings and physician discretion. Similar assessments were performed in symptomatic women without overt bleeding, but with a significant Hb drop, as estimated by the attending physician (with respect to the degree of Hb drop and the course of delivery). Intravenous iron sucrose (500 mg as a single dose) was administered in cases of Hb ≤ 9.5 g/dl. Iron sucrose + red blood cells were transfused in cases of Hb < 7 g/dl, regardless of symptoms or in cases of Hb < 8 with anemia-related symptoms.

### Interventions

Women included in the present analysis were divided into two groups:

1. PPH—CBC was performed either due to overt PPH or occult PPH.

2. Control—CBC was performed due to indications unrelated to bleeding or symptoms.

### Study endpoints

The primary endpoint was mean Hb decrease during the immediate postpartum period (during admission in the maternity ward). Secondary endpoints were Hb change over time from delivery and characteristics of women who required operative interventions while in the maternity ward. For women who received blood transfusions, Hb values were corrected by decreasing the elevation in Hb level following blood transfusion. In addition, the times at which Hb first fell below ≤ 9.5 g/dl and < 7 g/dl were identified. These cutoffs were chosen since they are considered indications for treatment with intravenous iron sucrose and blood transfusion + intravenous iron sucrose, respectively, according to our departmental protocol. The percent of women with first-documented Hb cutoffs was determined for each 6-h block from admission to the maternity ward.

### Statistical analysis

As mentioned above, this was a secondary analysis of data collected in a prospective cohort trial. The available sample size (N = 419) was sufficient to detect a 1.5 ± 4.0 g/dl Hb difference between the control and PPH groups (5% two sided alpha, 96% power). This calculation was chosen due to its clinical relevance. In addition, the sample size was sufficient to detect a ≥ 1.0 ± 2.5 g/dl Hb difference between the overt PPH, occult PPH and control groups (5% 2-sided alpha, at least 90% power).

Baseline characteristics and outcomes of the PPH and control cohorts were compared using the Student’s *t*-test (or Wilcoxon two sample test) for continuous variables and χ2 (or Fisher's exact test) for categorical variables.

The locally scatter plot smoothing (LOESS) non-parametric regression model was utilized to compare the mean Hb drop from pre-delivery measures over time from delivery (hours). 95% confidence intervals of the LOESS curves are also presented^9^.

Statistical analyses were carried out with SAS version 9.4 (SAS Institute, Cary, NC, USA). Significance was set at a *p* value of < 0.05.

## Results

**Figure 1:**
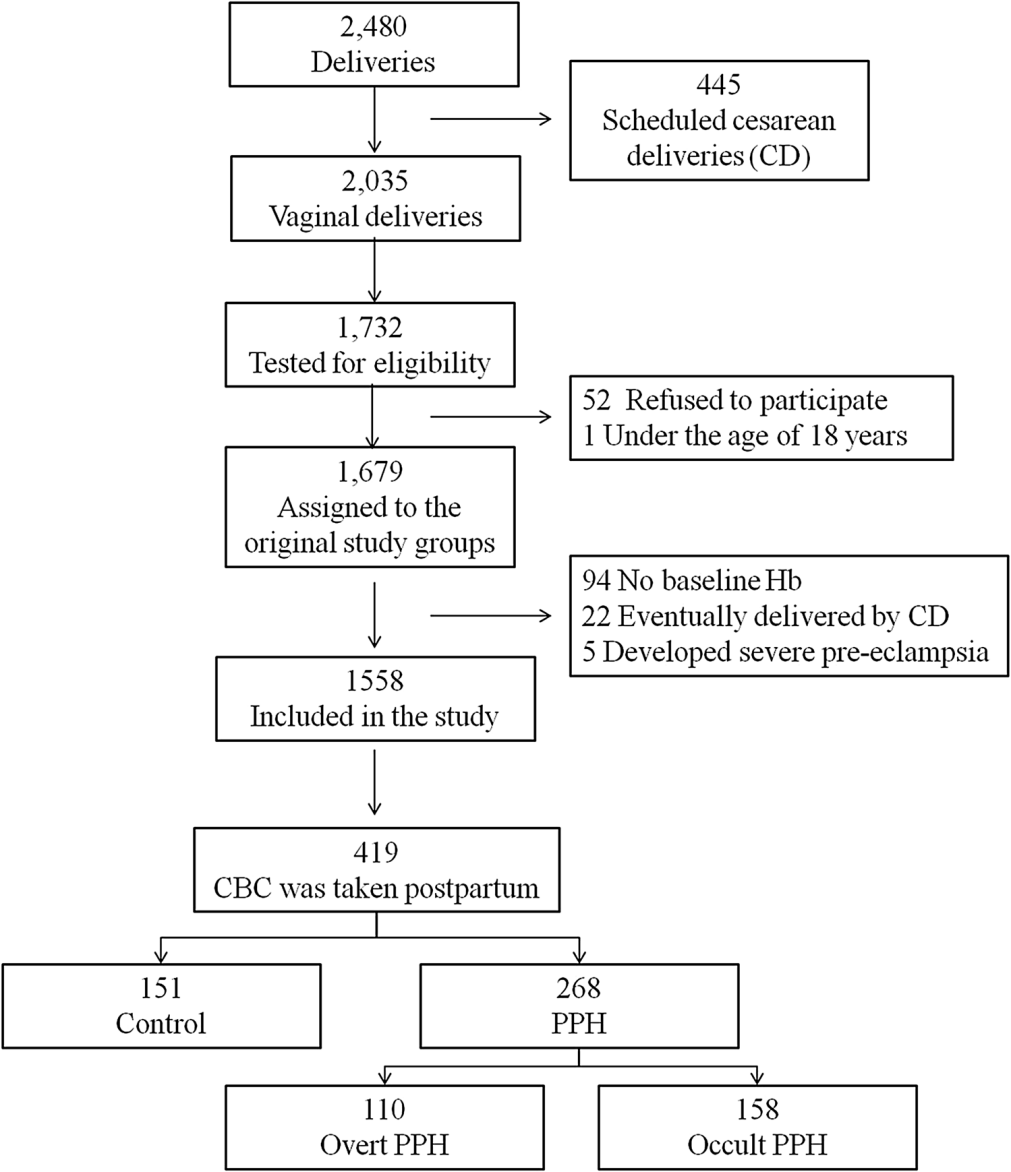
Patient disposition flow chart. *CBC* complete blood count, *CD* caesarean delivery, *Hb* hemoglobin, *PPH* postpartum hemorrhage.

Among the 1558 women who were included in the original study, a CBC was performed before and after delivery for 419 women; all these women were included in the present analysis. Of these, 268 (64%) women had bleeding-related reasons for CBC testing (PPH group) and 151 (36%) women had CBC monitored due to other indications (control group). In the PPH group, 158 (59%) presented with occult PPH and 110 (41%) presented with overt PPH. Among the 110 women with overt PPH, 38 (35%) had perineal, vaginal or cervical tears, 29 (26%) had retained product of conceptions, 34 (31%) had uterine atony and 9 (8%) had uterine atony and tear. A patient flow chart is presented in Fig. 1. Patient characteristics are summarized in Table 1. The PPH group had more cases of primiparity, perineal tears, episiotomy and manual exploration of the uterine cavity and cervix as compared to the control.

**Table 1:** Patient demographics, pregnancy, and delivery characteristics.

	Control (N = 151)	PPH (N = 268)	p value
Maternal age (y)	28.6 (5.5) [29, 25–32]	28.0 (5.1) [28, 24–31]	0.25
BMI (kg/m^2^)	24.2 (5.0) [23.2, 20.7–28.0]	24.0 (4.7) [23.2, 20.7–26.1]	0.74
Delivery num	2.6 (1.5) [2, 2–3]	1.9 (1.1) [1, 1–3]	< .0001
Primiparity	37 (25%)	137 (51%)	< .0001
Smoking	1 (1%)	1 (0.4%)	1
Hb before delivery (g/dl)	10.7 (1.4) [10.3, 9.6–11.9]	11.8 (1.3) [11.9, 11–12.7]	< .0001
HCT before delivery (%)	32 (4) [32, 29–35]	35 (4) [35, 33–37]	< .0001
Delivery week	39.1 (1.5) [39.2, 38.2–40.2]	39.4 (1.4) [39.5, 38.4–40.4]	0.03
Hemoglobinopathies	5 (3%)	4 (1%)	0.29
GDM	22 (15%)	29 (11%)	0.26
Pre-GDM	2 (1%)	3 (1%)	1
Chronic HTN	2 (1%)	2 (1%)	0.62
Gestational HTN	9 (6%)	17 (6%)	0.88
Clotting abnormalities	14 (9%)	16 (6%)	0.21
Epidural	50 (33%)	129 (48%)	0.003
Vaginal delivery	144 (95%)	241 (90%)	0.05
Vacuum	7 (5%)	27 (10%)	
Labor induction	63 (42%)	123 (46%)	0.41
Prolonged second stage	6 (4%)	20 (7%)	0.16
Shoulder dystocia	2 (1%)	3 (1%)	1
**Perineal tear grade**			
0	117 (77%)	160 (60%)	0.0002
1	20 (13%)	44 (16%)	
2	12 (8%)	59 (22%)	
3	1 (1%)	5 (2%)	
4	1 (1%)	0 (0%)	
Episiotomy	8 (5%)	51 (19%)	0.0001
Manual exploration of uterine cavity and cervix	4 (3%)	52 (19%)	< .0001
Manual lysis of placenta	3 (2%)	14 (5%)	0.11

Values are presented as mean (SD) (median, [interquartile range]) or number (percent).

*BMI* body mass index, *Hb* hemoglobin, *HCT* hematocrit.

Larger Hb and HCT reductions from pre-delivery measures were measured in the immediate postpartum period among women with PPH as compared to the control group (Table 2). A sub-analysis of women without pre-delivery anemia (Hb ≥ 10.5 g/dl; N = 66 and N = 227 in the control and PPH groups, respectively) showed a similar decrease in the control versus the PPH group of both Hb [1.1 ± 1.3 g/dl (median 0.85 g/dl, IQR 0.3–1.5 g/dl) versus 2.5 ± 1.6 g/dl (median 2.4 g/dl, IQR 1.3–3.6 g/dl), p < 0.0001; respectively] and HCT [3.3 ± 3.7% (median 2.6%, IQR 0.6–5.2%) versus 7.3 ± 4.6% (median 7.3%, IQR 3.6–10.7%), p < 0.0001; respectively]. A sub-analysis of the women with overt versus occult PPH showed that occult PPH was associated with a decline in Hb that fell between the decline measured in control and the decline measured in overt PPH patients (Table 3).

**Table 2:** Decrease in hemoglobin and hematocrit during the immediate postpartum period.

	Control (N = 151)	PPH (N = 268)	*p* value/OR_adjusted_ [95% CI]
Decrease of Hb (g/dl)	0.9 (1.0) [0.7, 0.2–1.3]	2.4 (1.6) [2.2, 1.2–3.4]	< 0.0001
Decrease of HCT (%)	3 (3) [3, 0.2–4]	7 (4) [7, 4–10]	< 0.0001
Decrease of HCT ≥ 10%	6 (4%)	65 (24%)	3.7 [1.5–9.2]
Decrease of Hb ≥ 2 g/dl	18 (12%)	148 (55%)	5.6 [3.1–10.2]
Decrease of Hb ≥ 3 g/dl	5 (3%)	91 (34%)	8.6 [3.3–22.3]

Values are presented as mean (SD) [median, interquartile range] or number (percent).

OR was adjusted for primiparity, epidural, perineal tears, episiotomy, Hb before delivery, HCT before delivery, delivery number and delivery week.

*Hb* hemoglobin, *HCT* hematocrit, *OR* odds ratio.

**Table 3:** Decrease in hemoglobin and hematocrit during the immediate postpartum period.

	Control (N = 151)	Occult PPH (N = 158)	p value/OR_adjusted_ [95% CI]	Overt PPH (N = 110)	p value/OR_adjusted_ [95% CI]*	p value/OR_adjusted_ [95% CI]**
Decrease of Hb (g/dl)	0.9 (1.0) [0.7, 0.2–1.3]	2.0 (1.4) [1.7, 1–2.8]	< 0.0001	3.0 (1.6) [2.9, 1.8–4.2]	< 0.0001	< 0.0001
Decrease of HCT (%)	3 (3) [3, 0.2–4]	6 (4) [5, 3–8]	< 0.0001	9 (4) [9, 6–12]	< 0.0001	< 0.0001
Decrease of HCT ≥ 10%	6 (4%)	21 (13%)	2.0 [0.8–5.4]	44 (40%)	7.3 [2.8–19.2]	3.6 [1.9–6.7]
Decrease of Hb ≥ 2 g/dl	18 (12%)	67 (42%)	3.7 [2.0–7.0]	81 (73%)	11.5 [5.6–23.3]	3.1 [1.7–5.5]
Decrease of Hb ≥ 3 g/dl	5 (3%)	36 (23%)	5.6 [2.1–15.0]	55 (50%)	15.4 [5.6–42]	2.8 [1.6–4.9]

Values are presented as mean (SD) [median, Interquartile range] or number (percent).

* Comparison between “Overt PPH” and “Control”.

** Comparison between “Overt PPH” and “Occult PPH”.

OR was adjusted for primiparity, epidural, perineal tears, episiotomy, Hb before delivery, HCT before delivery, delivery number and delivery week.

*Hb* hemoglobin, *HCT* hematocrit, *OR* odds ratio.

In total, 31 (2%) women received blood transfusions, 1 (0.7%) of whom belonged to the control group, 9 (6%) to the occult PPH group and 21 (19%) to the overt PPH group. The indication for blood transfusion in the patient in the control group was pre-delivery Hb of 6.1 g/dl. The Hb values following blood transfusions were corrected by decreasing the elevation in Hb level that was measured following blood transfusion; control [mean 0.9 ± 1 g/dl (median 0.7 g/dl, IQR 0.2–1.3 g/dl), occult PPH 2 ± 1.4 g/dl (1.7, 1–2.8 g/dl)] and overt PPH [3.2 ± 1.8 g/dl (3.2, 1.8–4.4 g/dl), p < 0.0001]. These results are similar to the results without correction (Table 2).

**Figure 2:**
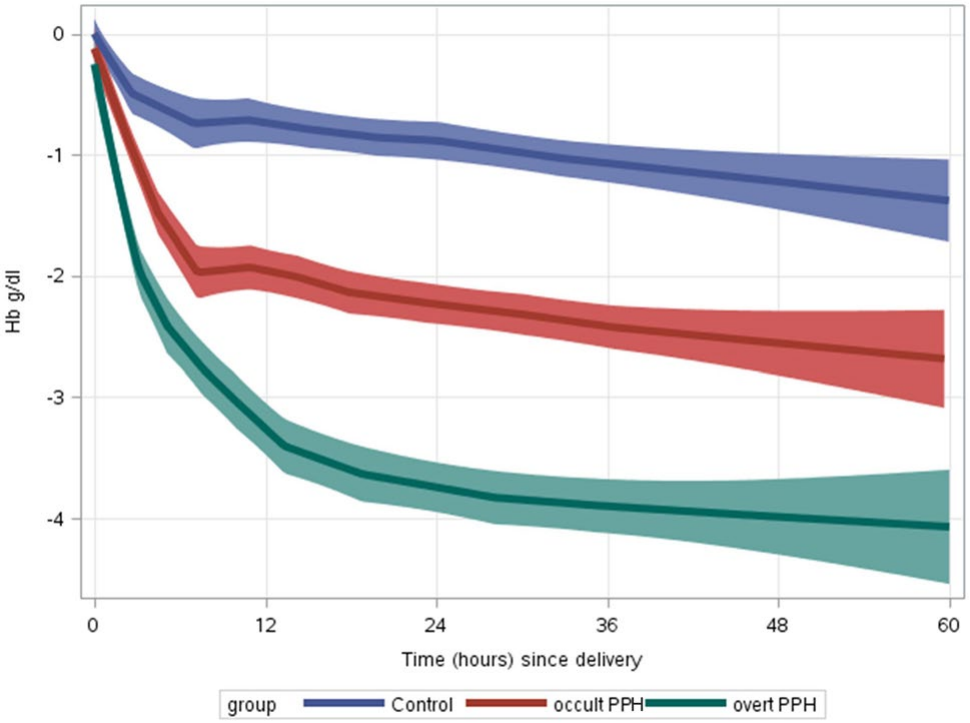
Hemoglobin drop during the immediate postpartum period. LOESS smooth curve (smoothing parameter 0.6) with 95% confidence interval of the mean hemoglobin (Hb) drop by time (hours) from delivery in women monitored due to overt postpartum hemorrhage (PPH), occult PPH or other indications (control). Correction for blood transfusion was made as described in the in the “Methods” section.

In order to compare the mean Hb drop during the post-partum period between the control, overt PPH and occult PPH groups, the LOESS non-parametric regression model was utilized. Correction of Hb values following blood transfusions as described above was done (Fig. 2). Maximal rate of decline in Hb values was witnessed during the first 6–12 h postpartum, followed by a more moderate rate of change between 12 and 24 h, which then plateaued after 24–48 h in all the groups, regardless of the absolute minimum value of Hb (Fig. 2). LOESS curves for HCT decrease and Hb decrease, without correction for blood transfusion, showed similar trends (data not shown).

**Figure 3:**
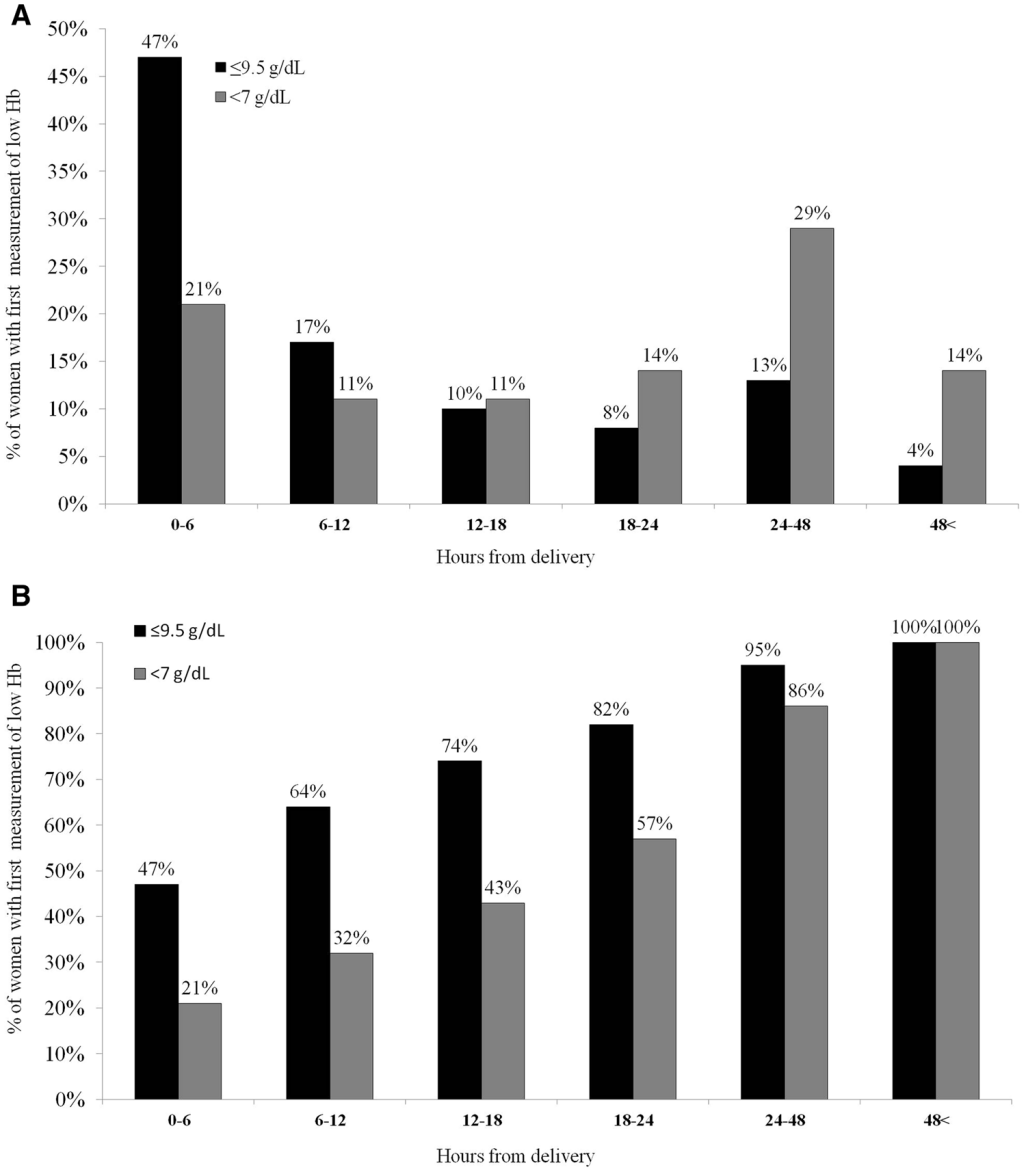
Time to Hb ≤ 9.5 g/dl or Hb < 7 g/dl. Percent **(A)** and cumulative percent **(B)** of women with first occurrence of hemoglobin (Hb) value either Hb ≤ 9.5 g/dl (N = 210) or Hb < 7 g/dl (N = 28), at and after delivery.

We examined the time when Hb dropped to ≤ 9.5 g/dl and < 7 g/dl for the first time. Those cutoffs were chosen since they are considered indications for treatment with intravenous iron sucrose and intravenous blood transfusion + intravenous iron sucrose, respectively, according to our departmental protocol. The results are presented as percent of women in blocks of 6 h at and after delivery and additional block beyond 48 h. Correction for blood transfusion was done as described above (Fig. 3). Among the women whose Hb levels eventually dropped to ≤ 9.5 g/dl (n = 210) and < 7 g/dl (n = 28), respectively, at 48 h postpartum, Hb was ≤ 9.5 g/dl and < 7 g/dl for 95% and 86% of the women, respectively. This suggests that follow-up of Hb drop should be for at least 48 h in cases of PPH.

Only ten of the 1558 women participating in the original study (0.6%) required operative intervention for PPH during their stay in the maternity ward. Seven (0.4%) had undergone a manual exploration of the uterine cavity and cervix due to overt hemorrhage. Two women (0.1%) underwent drainage of a vaginal hematoma that was discovered following tachycardia and significant perineal pain in one case, and urinary retention and significant perineal pain in the second case. One woman underwent a uterine cavity evacuation of blood clots due to overt hemorrhage and a large blood clot in uterine sonography that was not resolved after treatment with misoprostol. The LOESS curve of Hb drop of these women was similar to the curve of the overt PPH group (data not shown). The remaining operative interventions were performed in the delivery unit immediately after birth, to manage overt hemorrhage or hemodynamic instability. There were no cases of asymptomatic women who needed operative intervention in the maternity ward.

## Discussion

The present study aimed to explore the physiologic patterns of Hb and HCT following vaginal delivery in patients without PPH, or with occult or overt PPH. In women without PPH, mean Hb decrease was approximately 1 g/dl, while a 2 g/dl and 3 g/dl decrease was measured in women with occult PPH or overt PPH, respectively. A mean 3%, 6% and 9% HCT decrease was measured in women without PPH, or with occult or overt PPH, respectively. Sub-analysis of women without anemia before delivery showed similar results, suggesting that the degree of Hb and HCT decrease is more important than the absolute values, in inducing anemia/hypovolemia symptoms. The slope of Hb decrease was similar in all groups, which implies that this fall represents a late response to a hemorrhagic event that was terminated without active bleeding afterwards, with the exception of ten cases, who suffered active bleeding in the maternity ward. The LOESS curve demonstrated three Hb phases following delivery, i.e., a sharp decline in the first 6–12 h, followed by a moderate drop in the next 12–24 h, until stabilization after 24–48 h.

Although PPH is one of the most common complications of delivery and a leading cause of maternal death^7^, the behavior of Hb and HCT has never been the focus of a robust research. Most studies of Hb and HCT decrease after delivery have been in the context of overt PPH, where patient inclusion was based on visual estimation of increased hemorrhage after delivery^10^. However, addressing only cases of overt bleeding fails to assess the possible benefit of strict follow-up and treatment with iron sucrose and blood transfusion gained by women with occult PPH.

In 1991, Combs et al. defined PPH as a ≥ 10-point HCT decrease, a cutoff which corresponded to approximately the 97th percentile of HCT change in vaginal deliveries in their database^11^. In the present study, while this cutoff value corresponded to women with overt PPH, more subtle decreases in HCT values were sufficient to induce symptoms of hypovolemia. An additional study examined Hb decrease during PPH events after delivery by subtracting the Hb at time of discharge (median of 32 h after delivery) from the pre-delivery Hb. In cases where a red blood cell transfusion had been performed to maintain Hb > 8 g/dl, 1 g/dl for each unit of red blood cells transfused was added to the fall in Hb to give a corrected fall in Hb. The reported Hb decrease in cases of PPH was similar to those of the current PPH cohort. The decrease in Hb over time and Hb stabilization was not assessed in their study, as only one measurement was taken after delivery^12^.

Treatment with iron sucrose has gained popularity in the past few years, thanks to its superiority over oral iron supplementations in treating postpartum anemia^13^ and its high tolerability^8,13^. Current official guidelines recommend treating moderate to severe postpartum anemia (i.e., Hb ≤ 9.5 g/dl) with intravenous iron supplements, such as iron sucrose^14–16^. Understanding the physiologic behavior of Hb drop following delivery allowed us to provide recommendations for Hb monitoring postpartum, for the purpose of anemia detection and treatment. Previously*,* we demonstrated that routine screening of women with pre-delivery anemia for postpartum anemia led to increased anemia diagnosis and consequently better medical care^8^. Together with the results of the current study, we suggest that Hb levels should be determined after delivery in women with pre-delivery anemia (Hb < 10.5 g/dl) and in women with occult or overt PPH. Such monitoring will detect the majority of women who would benefit from iron sucrose treatment and blood transfusion.

The strengths of this study lay in the prospectively collected data, the high recruitment rate, which decreased selection bias, a large sample size , the use of a systematic protocol to follow Hb decrease until stabilization, and inclusion of both control and occult PPH groups. The limitations of this study included its nature as a secondary analysis, diagnosis of overt bleeding at staff discretion and not by accurate blood loss measurement, and the large percentage of women in the control group with pre-delivery anaemia, due to the design of the original study^8^. The latter limitation was overcome by performing a sub-analysis of women without pre-delivery anaemia and by adjusting the study endpoints for pre-delivery Hb and HCT. Another limitation of this study was absence of cases of intra-abdominal bleeding. In these rare cases, overt bleeding is not expected. However, it is likely that this event would be accompanied by symptomatic anemia and hemodynamic instability, both of which should be assessed for active bleeding, as was demonstrated in this study.

Taken together, laboratory results consistent with PPH are Hb decrease ≥ 2 g/dl and HCT decrease ≥ 6%. Post-partum symptoms of hypovolemia are a reliable marker for occult PPH, which represents an intermediate group between normal blood loss and overt PPH. Hb levels should be monitored for at least 48 h after delivery to detect anemia requiring iron sucrose treatment and/or blood transfusion. Finally, asymptomatic postpartum Hb drop usually does not suggest active bleeding.

## Data Availability

Data generated during this study is available upon a reasonable request and with accordance to the regulations of the institutional review board of Emek Medical Center.
